# Macro-ER-phagy receptors Atg39p and Atg40p confer resistance to aminoglycoside hygromycin B in *S. cerevisiae*

**DOI:** 10.17912/micropub.biology.000738

**Published:** 2023-01-31

**Authors:** Mahmoud M. Daraghmi, Jacob M. Miller, Connor G. Bailey, Ellen M. Doss, Ashley L. Kalinski, Philip J. Smaldino, Eric M. Rubenstein

**Affiliations:** 1 Department of Biology, Ball State University

## Abstract

Receptor-mediated autophagic turnover of portions of the endoplasmic reticulum (ER) is mediated by macro-ER-phagy. We hypothesized macro-ER-phagy promotes proteotoxic stress resistance. We predicted *Saccharomyces cerevisiae* lacking macro-ER-phagy receptors would exhibit enhanced sensitivity to hygromycin B, which reduces translational fidelity and is expected to globally disrupt protein homeostasis, including at the ER. We observed that loss of either of two yeast macro-ER-phagy receptors (Atg39p or Atg40p) compromised cellular resistance to hygromycin B to a similar extent as loss of ER-associated degradation (ERAD) ubiquitin ligases Hrd1p and Doa10p. Our data are consistent with a model whereby macro-ER-phagy and ERAD collaborate to mediate ER protein quality control. Disruptions of macro-ER-phagy have been linked to neuropathy, dementia, and cancer. A dampened capacity to mediate protein quality control may contribute to these conditions.

**Figure 1. ATG39 and ATG40 confer resistance to hygromycin B.**
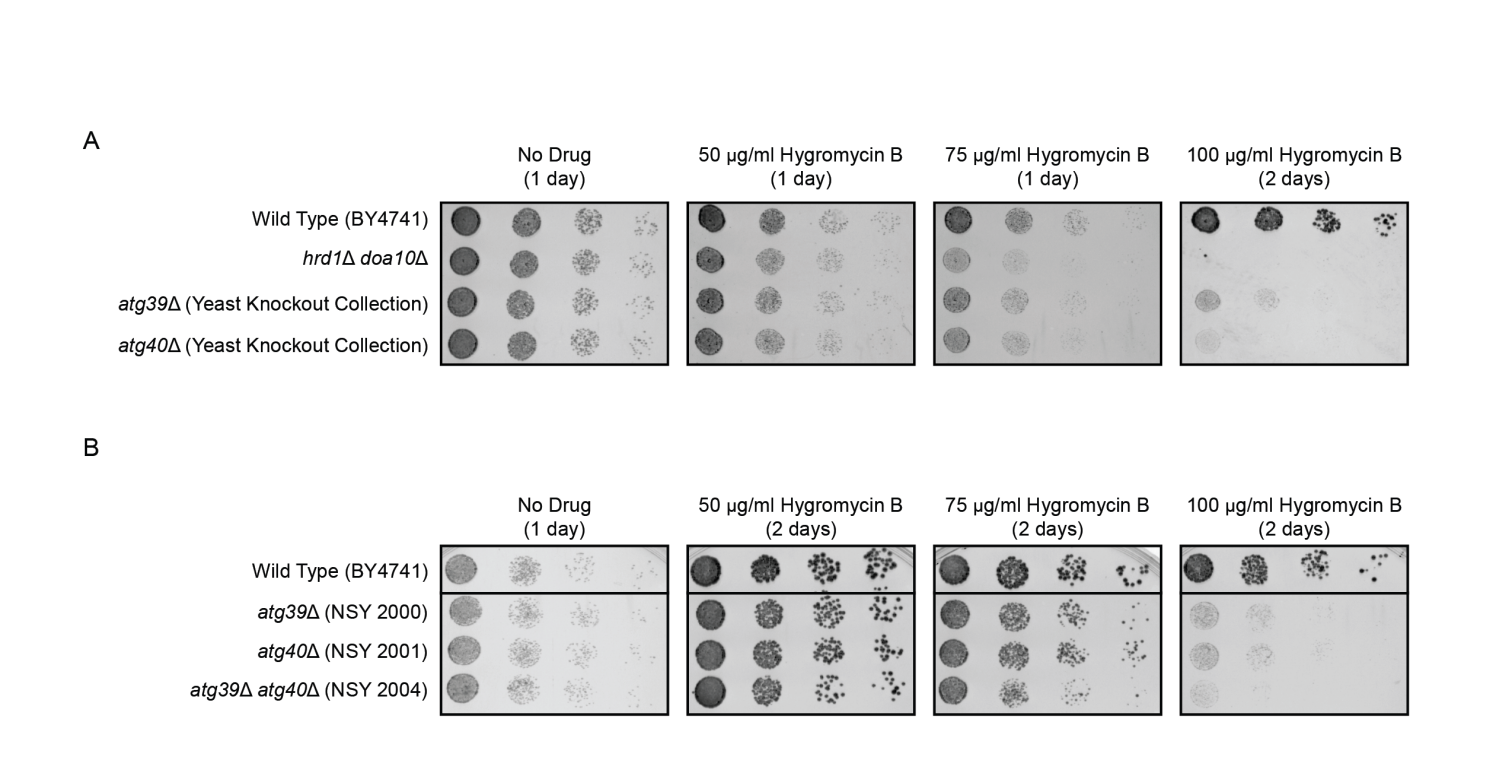
Serial dilutions of indicated yeast strains were spotted onto agar plates containing rich growth medium lacking or supplemented with increasing concentrations of hygromycin B. Plates were incubated at 30°C and imaged after 1-2 days. Experiments were performed in triplicate.

## Description

Degradation of proteins at the endoplasmic reticulum (ER) is mediated by ER-associated degradation (ERAD) and ER autophagy (ER-phagy). In ERAD, ubiquitin ligases mark individual ER-localized proteins with ubiquitin for proteasomal degradation (reviewed in (Christianson and Carvalho, 2022; Mehrtash and Hochstrasser, 2019)). By contrast, ER-phagy involves the lysosomal destruction of larger portions of ER (reviewed in (Mochida and Nakatogawa, 2022; Yang et al., 2021)). ER-phagy contributes to ER homeostasis under a range of conditions, including ER stress and nutrient limitation (Bernales et al., 2007; Houck et al., 2014; Khaminets et al., 2015; Lipatova and Segev, 2015; Mochida et al., 2015; Nakatogawa and Mochida, 2015; Schuck et al., 2014).

Multiple unique forms of ER-phagy exist with distinct regulation and genetic requirements. In micro-ER-phagy, ER segments are directly degraded by the lysosome (called the vacuole in yeast), independently of characterized core autophagy-related (Atg) proteins and autophagosome formation. Micro-ER-phagy is stimulated by ER stress (elevated abundance of misfolded ER proteins) (Schuck et al., 2014). By contrast, macro-ER-phagy requires the core autophagic machinery. In macroautophagy, unique receptor proteins interact with organelle-specific cargo at the surface of an organelle (Khaminets et al., 2015; Lipatova and Segev, 2015; Mochida et al., 2015). This interaction allows receptor proteins to associate with the phagophore, a double membrane that surrounds the compromised organelle and closes to form an autophagosome (Khaminets et al., 2015; Mochida et al., 2015). Autophagosomes fuse with lysosomes to form autophagolysosomes, wherein encapsulated contents are degraded (Berg et al., 1998; Itakura et al., 2012; Takats et al., 2013). Macro-ER-phagy promotes vacuolar degradation of excess integral membrane proteins (Lipatova and Segev, 2015) and reduces accumulation of aggregation-prone ER proteins (Cui et al., 2019).

Two proteins, Atg39p and Atg40p, serve as macro-ER-phagy receptors in *Saccharomyces cerevisiae* . Atg39p facilitates turnover of perinuclear ER, while Atg40p promotes cytoplasmic and cortical macro-ER-phagy (Mochida et al., 2015). The mammalian homolog of Atg40p (FAM134Bp) enables ER-phagy by a similar mechanism (Khaminets et al., 2015). *ATG39* / *ATG40* expression and Atg39p/Atg40p-dependent macro-ER-phagy are induced by ER stress, nitrogen starvation, and rapamycin (Cui et al., 2019; Mizuno et al., 2020; Mochida et al., 2015). Whether macro-ER-phagy is required under conditions of global proteotoxic stress has not been determined. We determined whether *ATG39* and *ATG40* promote resistance to hygromycin B, an aminoglycoside produced by *Streptomyces hygroscopicus* . Hygromycin B distorts the ribosome aminoacyl site, thereby reducing translational fidelity, and is expected to broadly perturb the cellular proteome (Brodersen et al., 2000; Ganoza and Kiel, 2001). We and others have previously shown several ER and nuclear ubiquitin ligases and associated proteins are required for optimal growth in the presence of hygromycin B (Bengtson and Joazeiro, 2010; Crowder et al., 2015; Doss et al., 2022; Runnebohm et al., 2020a; Verma et al., 2013; Woodruff et al., 2021). Given that approximately one third of eukaryotic proteins are imported into the ER en route to their subcellular or extracellular destination, we hypothesized that macro-ER-phagy collaborates with ER and nuclear ubiquitin ligases to promote survival during proteotoxic stress.

To investigate potential general contributions of Atg39p and Atg40p to protein quality control, we cultured wild type yeast, yeast lacking genes encoding both primary ERAD ubiquitin ligases, *DOA10* and *HRD1* (Buchanan et al., 2019), and yeast lacking either *ATG39* or *ATG40* in the absence or presence of increasing concentrations of hygromycin B (Figure 1A). The *atg39* Δ and *atg40* Δ strains were taken from the Yeast Knockout Collection (Giaever and Nislow, 2014). All strains exhibited similar growth in drug-free media. As previously observed (Crowder et al., 2015; Niekamp et al., 2019), *hrd1* Δ *doa10* Δ yeast exhibited markedly reduced growth relative to wild type cells with increasing hygromycin B concentrations. Deletion of *ATG39* or *ATG40* also sensitized yeast to hygromycin B, consistent with our hypothesis that macro-ER-phagy mitigates stress associated with a broadly disrupted proteome. To validate the observation that ER-phagy receptor loss impairs hygromycin B resistance, we evaluated growth of independently generated single and double knockout yeast lacking *ATG39* and/or *ATG40* (Lipatova et al., 2020). Consistent with data in Figure 1A, these *atg39* Δ and *atg40* Δ yeast exhibited impaired growth in the presence of hygromycin B; simultaneous loss of both genes modestly further enhanced drug sensitivity (Figure 1B).

Our results demonstrate that macro-ER-phagy receptors are required for maximal resistance to the aminoglycoside hygromycin B. Modestly enhanced hygromycin B sensitivity of *atg39* Δ *atg40* Δ relative to either single mutant is consistent with Atg39p and Atg40p possessing partially overlapping function, which may be expected given the physical continuity and partially overlapping proteome of the ER and nuclear envelope (Deng and Hochstrasser, 2006). Indeed, previous reports indicate contribution of both Atg39p and Atg40p to turnover of the same model macro-ER-phagy substrates (Mizuno et al., 2020; Mochida et al., 2015).

A role for Atg39p and Atg40p in combatting proteotoxic stress is consistent with quality control functions of macro-ER-phagy receptors suggested by other reports (Cui et al., 2019; Lipatova et al., 2020; Mizuno et al., 2020). Additional studies will be required to determine whether (and by what mechanism) Atg39p and Atg40p abundance increases in the face of proteotoxic stress (as occurs following ER stress, nitrogen limitation, and rapamycin treatment (Cui et al., 2019; Mizuno et al., 2020; Mochida et al., 2015). Further, analyses of macro-ER-phagy-defective mutants cultured in the presence of drugs that perturb proteostasis by different mechanisms may better define the range of stress conditions against which Atg39p and Atg40p protect. Consistent with a role in protein quality control for macro-ER-phagy, a negative genetic interaction between mutations of *ATG40* and *STE24* (which encodes a zinc metalloprotease with ER protein quality control function (Ast et al., 2016; Runnebohm et al., 2020b)) has been detected in a large-scale interaction study (Costanzo et al., 2016). Future experiments may be conducted to directly assess the overlap between macro-ER-phagy and ERAD in mitigating proteotoxic stress. Macro-ER-phagy defects are associated with a number of ailments in humans and mammalian research models, including neuropathy, dementia, and cancer (Haque et al., 2016; Islam et al., 2017; Khaminets et al., 2015; Kong et al., 2011; Kurth et al., 2009). The extent to which impaired quality control contributes to the development of these conditions remains to be determined.

## Methods

**Yeast growth assays.** Yeast growth experiments were performed as described (Watts et al., 2015). Four μl of sixfold serial dilutions were transferred to agar plates containing yeast extract-peptone-dextrose (YPD) medium (Guthrie and Fink, 2004) lacking or possessing hygromycin B (Gibco). Plates were incubated at 30°C and imaged on the indicated days.

## Reagents

**Table:** 

**Name**	**Genotype**	**Reference**
VJY305 (alias SKY252)	*MATa his3* Δ *1 leu2* Δ *0 met15* Δ *0 ura3* Δ *0 hrd1* Δ *::kanMX4 doa10* Δ *::kanMX4*	(Habeck et al., 2015)
VJY421	*MATa his3* Δ *1 leu2* Δ *0 met15* Δ *0 ura3* Δ *0 atg39* Δ *::kanMX4*	(Tong et al., 2001)
VJY422	*MATa his3* Δ *1 leu2* Δ *0 met15* Δ *0 ura3* Δ *0 atg40* Δ *::kanMX4*	(Tong et al., 2001)
VJY476 (alias BY4741)	*MATa his3* Δ *1 leu2* Δ *0 met15* Δ *0 ura3* Δ *0*	(Tong et al., 2001)
VJY1054 (alias NSY2000)	*MATa his3* Δ *1 leu2* Δ *0 met15* Δ *0 ura3* Δ *0 atg39* Δ *::kan*	(Lipatova et al., 2020)
VJY1055 (alias NSY2001)	*MATa his3* Δ *1 leu2* Δ *0 met15* Δ *0 ura3* Δ *0 atg40* Δ *::kan*	(Lipatova et al., 2020)
VJY1056 (alias NSY2004)	*MATa his3* Δ *1 leu2* Δ *0 met15* Δ *0 ura3* Δ *0 atg39* Δ *::nat atg40* Δ *::kan*	(Lipatova et al., 2020)
